# Information Recovery in Behavioral Networks

**DOI:** 10.1371/journal.pone.0125077

**Published:** 2015-05-06

**Authors:** Tiziano Squartini, Enrico Ser-Giacomi, Diego Garlaschelli, George Judge

**Affiliations:** 1 Istituto dei Sistemi Complessi, Universitá di Roma “Sapienza”, 00185 Rome, Italy; 2 IFISC (CSIC-UIB), Instituto de Física Interdisciplinar y Sistemas Complejos, Campus Universitat des les Illes Balears, E-07122 Palma de Mallorca, Spain; 3 Lorentz Institute for Theoretical Physics, University of Leiden, 9506 Leiden, Netherlands; 4 Graduate School and Giannini Foundation, University of California, Berkeley, CA 94720, United States; National Scientific and Technical Research Council (CONICET)., ARGENTINA

## Abstract

In the context of agent based modeling and network theory, we focus on the problem of recovering behavior-related choice information from origin-destination type data, a topic also known under the name of network tomography. As a basis for predicting agents' choices we emphasize the connection between adaptive intelligent behavior, causal entropy maximization, and self-organized behavior in an open dynamic system. We cast this problem in the form of binary and weighted networks and suggest information theoretic entropy-driven methods to recover estimates of the unknown behavioral flow parameters. Our objective is to recover the unknown behavioral values across the ensemble analytically, without explicitly sampling the configuration space. In order to do so, we consider the Cressie-Read family of entropic functionals, enlarging the set of estimators commonly employed to make optimal use of the available information. More specifically, we explicitly work out two cases of particular interest: Shannon functional and the likelihood functional. We then employ them for the analysis of both univariate and bivariate data sets, comparing their accuracy in reproducing the observed trends.

## 1 Introduction

In this paper we focus on the problem of recovering behavior-related micro choice information from aggregate data. In particular, we consider origin-destination data, casting this problem as an inference problem concerning the prediction of flows on networks [1–4]. We recognize that this type of data comes from dynamic, adaptive behavior systems involving interdependent micro components which give rise to an instantaneous, feedback-adaptive, world: as a result, such systems are non-deterministic in nature, involve information and uncertainty and are driven toward a certain, optimal, stationary state (see, for example, [5, 6]). As a basis for predicting agents’ choices, we cast this as a self-organized, equilibrium seeking system in the form of weighted and binary networks; we make use of information theoretic entropy-based methods to solve the ill-posed stochastic inverse problem and recover estimates of the unknown binary parameters.

### 1.1 Binary Network Problem

To go beyond traditional reductionist modeling and mathematical anomalies, we use a new paradigm that is developing under the name of Network Science (see, for example, [7, 8] and the references contained therein). There are several things that make this approach attractive for information recovery in economics and in other social sciences: for example, in the economic-behavioral sciences everything seems to depend on everything else and this fits right into the interconnectedness simultaneity of the nonlinear (and dynamic) network paradigm. Another example is provided by microeconomic theory, where the network representation of markets arises quite naturally (in fact, in many ways markets and binary networks are equivalent—see [9]). Finally, in terms of a methodology, network problems are consistent with the information theoretic approach to information recovery (see [10, 11]).

We seek an expression for the probabilities that the origin and the destination nodes are connected along a specific pathway in the statistical ensemble of possible pathways, without explicitly sampling the configuration space. Given information about the origin-destination network structure in the form of a matrix **A**, the unknown pathway probabilities *p*_*ij*_ must be estimated from aggregate flow data that may be noisy in nature. The number of unknown pathway parameters of the protocol matrix **A** is much larger than the number of measured aggregate origin-destination data points and, moreover, the components of the matrix **A** cannot be observed directly. This means that although the observed data are considered to be directly influenced by the values of model components, the observations only indirectly reflect the influence of the latter: as a result, the analyst must use indirect noisy observations to recover information on the unobserved vector of parameters. As a consequence, the relationship characterizing the effect of unobservable components on the observed data must be somehow inverted. This type of ill posed pure or stochastic inverse regularization problem cannot be solved by traditional econometric information recovery methods.

### 1.2 Status Measure

As we seek new ways to think about the causal adaptive behavior of complex and dynamic micro systems, we note that problems of this type may be re-formulated as problems of constrained entropy-maximization over the pathways. In other words, causal entropy maximization can be adopted as the systems status-measure and optimization criterion (following [12]). The result provides an exact expression for the occurrence of the unknown probabilities over the ensemble of pathways and yields the preferred probability distribution (see [13]).

This permits us to recast a behavioral system in terms of path microstates where entropy reflects the number of ways a macrostate can evolve along a path of possible microstates: the more diverse the number of path microstates, the larger the causal path entropy. The result is a causal entropic force that captures self-organized equilibrium seeking behavior (see [12, 14]). In other words, *causal entropy maximization is a link that leads us to believe that a behavioral system with a large number of individuals, interacting locally and in finite time, is in fact optimizing itself*. We would like to stress that the optimization tendency characterizing behavioral systems is what qualifies entropy-based inference methods as the most correct ones to model such systems. The rationale beyond this lies in the nature of their adaptive behavior: agents tend to adapt behavior in line with an optimizing principle (as the maximization of the future, accessible paths diversity—also definable, more generally, as “resources” [12, 13]), whence the need for a robust estimation procedure making the best use of the available information while disergarding any other arbitrary assumption. On the contrary, most behavioral economic-econometric models rest upon *ad hoc* assumptions which may lead to the identification and biased estimates of the unknown parameters, the underlying inference procedure and, in turn, the conclusions about the agents’ behavior (see [15–17]).

In the sections ahead we analyse systems within this framework, that permits the interpretation of adaptive economic behavior in terms of entropic functions: as a basis for solving micro-behavioral information recovery problems, we suggest an information theoretic family of entropic functions; to demonstrate applicability, we consider binary and weighted data sets and recover the optimum corresponding unknown probabilities.

## 2 Information Recovery Framework

In developing a basis for the use of information theoretic (IT) methods to infer origin-destination networks flows, we focus on a stochastic ill posed inverse problem and the corresponding regularization method it implies (the pure, without-noise inverse problem is just a special case). In this context the Cressie-Read (CR) family of entropic functions [18, 19] provides a basis for linking the data and the unknown model parameters.

This permits the researcher to exploit the statistical machinery of information theory to gain insights on the underlying adaptive behavior of a dynamic process from a system that may not be in equilibrium. This approach contrasts with the traditional approach to micro information recovery that rests on reductionist economic and econometric functional analysis and observational agent behavior data: however, precisely because of the nonlinear and ordinal nature of dynamic micro systems, the traditional approach is cumbersome in terms of identifying and expressing adaptive behavior.

We start introducing the CR multi parametric convex family of entropic functional measures [20]:

$$\begin{array}{c} I\left(p,q,\gamma \right)=\frac{1}{\gamma (\gamma +1)}\sum_{c}p_{c}[{\left(\frac{p_{c}}{q_{c}}\right)}^{\gamma }-1]. \end{array}$$ (1)

In Eq 1, *γ* is a parameter that indexes members of the CR family, *p*_*c*_’s represent the subject probabilities and the *q*_*c*_’s are interpreted as reference (or prior) probabilities (the reason for indexing our coefficients with *c* will be clarified in the following section). Being probabilities, the usual properties of *p*_*c*_, *q*_*c*_ ∈ [0, 1], ∀*c*, and ∑_*c*_ *p*_*c*_ = 1, ∑_*c*_ *q*_*c*_ = 1 are assumed to hold. As *γ* varies the resulting CR estimator that minimize the divergence between **p** and **q** exhibits a qualitatively different behavior that includes, as noteworthy examples, the Kullback-Leibler measure (in the limit as *γ* → 0 as Shannon entropy and in the limit as *γ* → −1 as the likelihood functional) and, in a binary context, the logistic distribution-divergence (see [21]).

In other words, the CR family of power divergences is a class of additive convex functions that encompasses a broad family of test statistics, in turn representing a broad family of functional relationships within a moments-based estimation context. In addition, the CR measure exhibits proper convexity in **p**, for all values of *γ* and **q**, and embodies characteristics such as additivity and invariance with respect to a monotonic transformation of the divergence measures. In the context of extremum metrics, the CR family represents a flexible family of pseudo-distance measures from which to derive empirical probabilities.

## 3 Integer Versions of the CR Family

In what follows we consider the two values *γ* = −1, 0, corresponding respectively to the *likelihood functional* and the *Shannon functional*. In the limit as *γ* → 0

$$\begin{array}{c} \lim_{\gamma \to 0}I\left(p,q,\gamma \right)=\sum_{c}p_{c}\ln(\frac{p_{c}}{q_{c}}) \end{array}$$ (2)

the Kullback-Leibler divergence between **p** and **q** is obtained. The particular case of a uniform prior, *q*_*c*_ = 1/*C*, allows us to recover the usual form of (minus) the *Shannon entropy* of the **p** distribution: $I\left(\text{p},\frac{1}{C},0\right)=\sum_{c}p_{c}\text{ln}p_{c}+\text{ln}C$. In the limit as *γ* → −1 provides the second functional of our list

$$\begin{array}{c} \lim_{\gamma \to -1}I\left(p,q,\gamma \right)=\sum_{c}q_{c}\ln(\frac{q_{c}}{p_{c}}) \end{array}$$ (3)

the Kullback-Leibler divergence between **q** and **p**. The particular case of uniform prior *q*_*c*_ = 1/*C* allows us to recover the usual form of (minus) the *likelihood function* of the **p** distribution: $I\left(\text{p},\frac{1}{C},-1\right)=-\sum_{c}\frac{\text{ln}p_{c}}{C}-\text{ln}C$.

We stress that while the Shannon functional has been already employed for the analysis of univariate and bivariate data sets, the likelihood functional case has not been explicitly worked out yet, thus representing the major contribution of this paper to the analysis of behavioral networks.

## 4 Network Behavior Recovery

**Fig 1:**
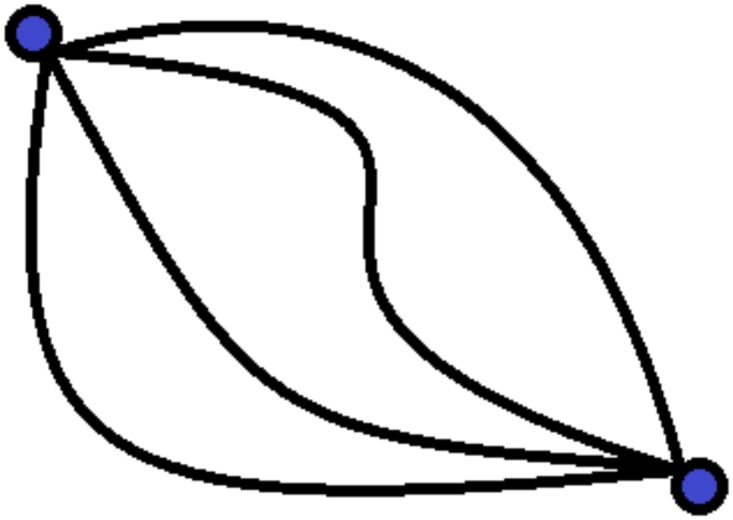
A schematic representation of an origin-destination network. Blue dots represent the origin and the destination nodes. Connections between them represent the ensemble of pathways described by the probability distribution ${\left\{p_{c}\right\}}_{c=1}^{C}$. The CR family allows one to determine the probability coefficients *p*_*c*_, ∀*c* by making use of the available partial information, i.e. aggregate data on traffic volumes.

To demonstrate the applicability of our approach in the binary network area, an example may be useful. Consider the problem of determining least-time, point-to-point traffic flows between sub-networks, when only aggregate origin-destinations volumes are known (see Fig 1). In many ways this is like a transportation network, with the emphasis on design and efficiency in routing the traffic flows (see [2] and the references therein), exactly as in an economic-behavioral network the efficiency of information flow is predicated on discovering, or designing, protocols that efficiently route information. The research question concerns the prediction of the volume of flows on the pathways, given a set of measures taken along them.

If we indicate by **y** the *R*-dimensional vector of observed fluxes and by **x** the *C*-dimensional vector of intermediate measures, the “activity” of an origin-destination network can be summed up by writing

$$\begin{array}{c} y=Ax \end{array}$$ (4)

where **A** is an *R* × *C* rectangular matrix, encoding the information about connections. Thus, our problem translates into estimating **x** on the basis of the *R*, available components of **y** and the connection structure **A**. The ill-posed nature of the problem is such that the inversion of Eq 1 is not feasible: the number of unknowns is greater than the number of known data, i.e. *R* < *C*. In this case, one can resort to the information theoretic methodology for solving problems of inference on the basis of partial information (see [22–25]). In order to implement, the problem unknowns have to be interpretable as probabilities and estimated on the basis of some known distribution moments. In our case, this can be easily achieved by dividing both sides of Eq 1 by *x*_*tot*_ ≡ ∑_*c*_ *x*_*c*_:

$$\begin{array}{c} \frac{y}{x_{tot}}\equiv r=Ap\equiv A\frac{x}{x_{tot}} \end{array}$$ (5)

where **y** and **A** are known, **p** is unknown and ∑_*c*_ *p*_*c*_ = 1. We have thus rewritten Eq 1 in terms of *fractions of fluxes* distributed across the *C* channels and interpret them as unknown probabilities. Notice that this peculiar definition of probability coefficients induces a distribution on the set of pathways, that play the role of an *ensemble* and allows us to restate the problem of predicting the fluxes on origin-destination networks as a (more) general problem of statistical inference. We can now may make use of the CR family of entropic divergence measures and write the problem as the following constrained optimization problem:

$$\begin{array}{c} 𝓛\equiv I\left(p,q,\gamma \right)-\theta_{0}[\sum_{c}p_{c}-1]-\sum_{\alpha }\theta_{\alpha }[\sum_{c}p_{c}A_{\alpha c}-r_{\alpha }] \end{array}$$ (6)

In particular, since the functional *I* is a divergence, the Lagrangean function has to be minimized with respect to the vector of coefficients **p**. This gives us the desired coefficients ${\left\{p_{c}\right\}}_{c=1}^{C}$ as functions of the Lagrangean multipliers, $p_{c}=p_{c}\left(\vec{\theta }\right),\;\forall \;c$. Once found, the parametric probability coefficients must be substituted back into 𝓛, in order to obtain a quantity which is a function of the unknowns solely: $𝓛(\vec{\theta })$. The last step of our procedure prescribes the optimization of the function $𝓛(\vec{\theta })$.

A similar problem is faced whenever a whole matrix of probability coefficients (and not a simple vector), **P**, is considered. Problems of this type can be formulated in much the same way, by writing the equation

$$\begin{array}{c} y^{\prime }=x^{\prime }P \end{array}$$ (7)

thus mimicking Eq 1. As we will show, treating **y**′ and **x**′ as known vectors allows us to succesfully also tackle this second type of problem.

These are just the solutions to a standard problem when a function must be inferred from insufficient sample-data information. Thus network inference and monitoring problems have a strong resemblance to an inverse problem in which key aspects of a system are not directly observable (for details on the use of information theoretic entropic methods for this type of network information flow problems see also [23–26]).

## 5 Applications

To test the effectiveness of our method, in what follows we analyze two aggregate data sets (for which origin-destination traffic volumes were collected), the first one concerning traffic on a local area network and the second one concerning consumers’ choices of complementary products.

**Fig 2:**
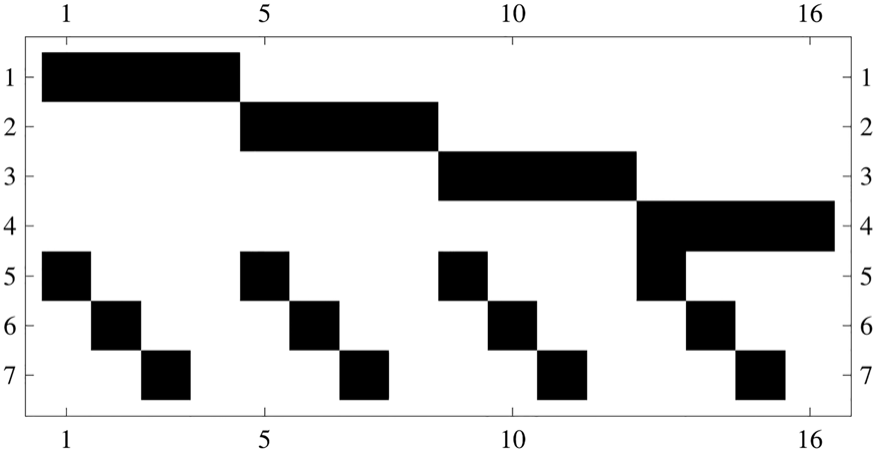
Pictorial matrix representation of a local area network at Bell Labs (black squares represent ones, white squares represent zeros—see [23, 27]), composed by four subnetworks (fddi, corp, local and switch) communicating via a router. The network topology we consider yields 7 observed aggregate traffic volumes and 16 origin-destination traffic volumes.

**Bell Labs data.** The first data set involves traffic volumes on a local area network at Bell Labs (see [23, 27]) whose routing matrix is reported in Fig 2. The network topology we consider here yields 7 observed aggregate traffic volumes and 16 origin-destination traffic volumes to be estimated. Aggregate volumes were measured every five minutes, over one day, on the Bell Labs corporate network, resulting in a set of measurements of 287 time points (see **Figures A** and **B** in S1 Information for another application of our method to univariate data sets).

**Complementary products.** The second data set comes from an economic case-study and relates to consumers’ behavior in the purchase of eggs and bacon (see [23, 28]). In particular, data consist of a sample of 548 independent households and the purchased products at the market, recorded over 4 consecutive trips. For each trip, it was recorded whether or not the household purchased eggs, bacon or both: the matrix entries represent the number of times a given customer purchased bacon and eggs over the course of the 4 trips, as reported in Table 1 [28] (see **Tables A** and **B** in S1 Information for another application of our method to bivariate data sets).

**Table 1:** Observed bivariate distribution of the number of times bacon and eggs were purchased on four consecutive shopping trips (see [23, 28]).

	Eggs					
Bacon	0	1	2	3	4	Total
0	254	115	42	13	6	430
1	34	29	16	6	1	86
2	8	8	3	3	1	23
3	0	0	4	1	1	6
4	1	1	1	0	0	3
Total	297	153	66	23	9	548

### 5.1 Bell Labs data

**Fig 3:**
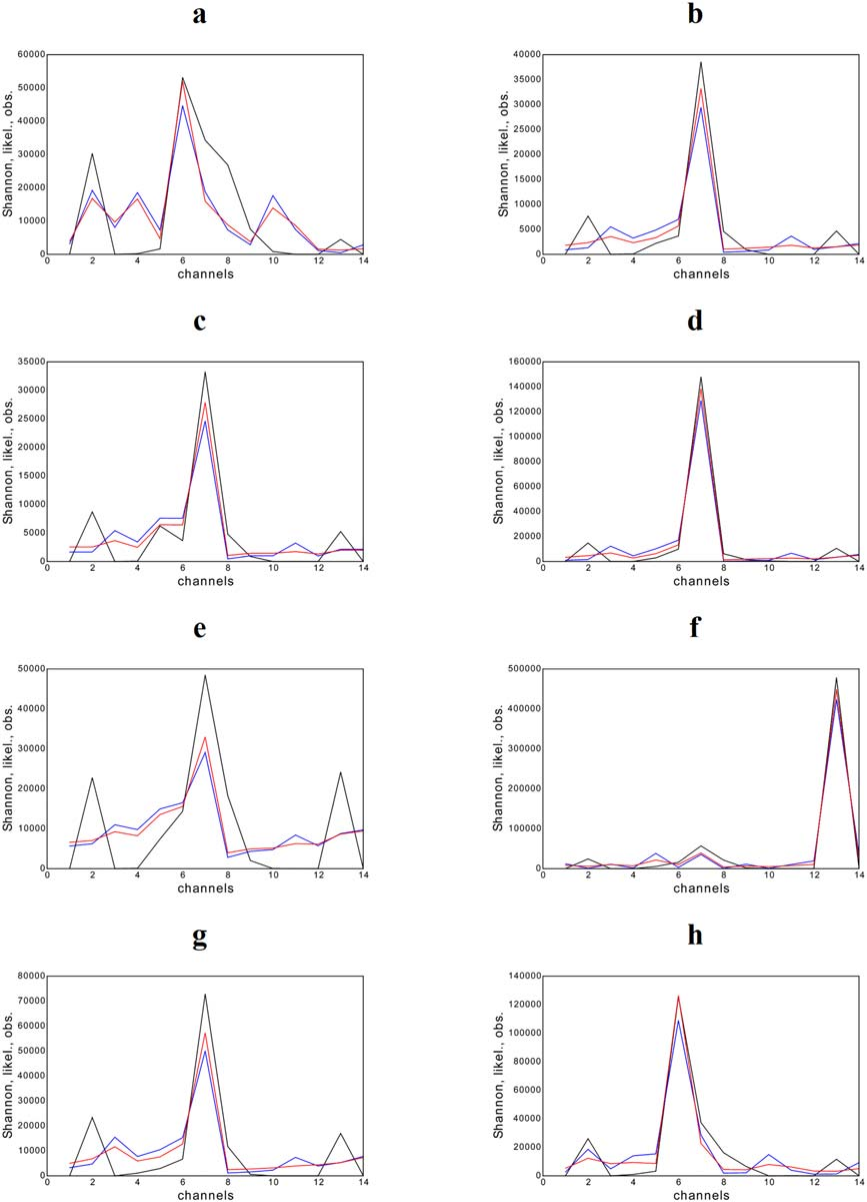
Analysis of Bell Labs data corresponding to ten chosen time points. The number of the channel is reported on the x-axis. Observed and estimated **x** are reported on the y-axis. Colors refers to: observed data (black trend), our estimation based on Shannon functional (blue trend), our estimation based on the likelihood functional (red trend).

**Fig 4:**
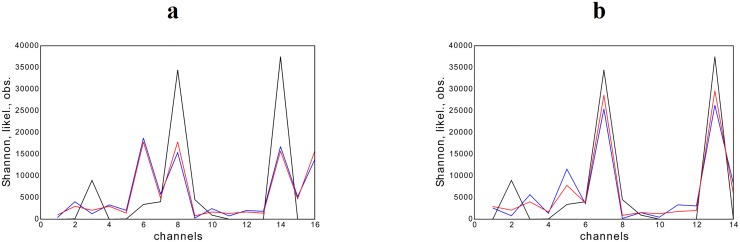
Analysis of Bell Labs data for the 90th time point. The number of the channel is reported on the x-axis. Observed and estimated **x** are reported on the y-axis. Colors refers to: observed data (black trend), our estimation based on Shannon functional (blue trend), our estimation based on the likelihood functional (red trend). Left panel: zero traffic flows are included in the data set. Right panel: zero traffic flows are excluded from the data set.

The analysis of Bell Labs data is illustrated in Figs 3 and 4. The panels report what we have called “channel plots”, showing the label of each origin-destination pattern (or channel) on the x-axis and the traffic volumes measured and estimated on it, on the y-axis. Black trends represent the observed traffic volumes and colored trends represent the expected traffic volumes, predicted via our procedure: blue trends represent the predictions obtained by using Shannon functional, red trends represent the predictions obtained with the empirical likelihood functional. Each panel corresponds to a given time point, chosen among the 287 available possibilities.

As a general comment, the predictions of both functionals reproduce the majority of the observed trends satisfactorily, with the likelihood functional performing slightly better than Shannon functional whose estimates, in some cases, show larger discrepancies. Moreover, the performance of both functionals improves when single peaks are registered on a single channel, accompanied by small traffic volumes on the others. However, at night, whenever the latter are exactly zero the agreement between our estimates and observations seems to deteriorate: as shown in the left panel of Fig 4, if zero traffic flows happen to be measured on some line, both Shannon and the likelihood functionals predict smaller peaks and larger values for the neighboring lines.

A solution to improve the predictions accuracy is to explicitly exclude zero values from our dataset. This can be achieved by considering a reduced **x** vector and a reduced **A** matrix without the 1st and the 16th columns, i.e. precisely those contributing to the values *x*_1_ = *x*_16_ = 0. The right panel of Fig 4 shows how much the accuracy of our method is improved: notice how peaks are reproduced much better now and traffic values on the neighboring lines are predicted to be much smaller than the former, as observed values confirm. The predicted trends in Fig 3 are calculated by adopting the same criterion, i.e. explicitly excluding the zero values on the extreme channels.

### 5.2 Complementary products

The result of the application of our information recovery method to the “eggs and bacon” data set is shown in Table 2. Since the anaysis concerns a bivariate network, the predictions of our functionals concern the matrix entries, estimated from the available rows and columns totals (see the S1 Information, “Bivariate data sets—SI” section, for the detailed calculations).

**Table 2:** Expected bivariate distribution of the number of times bacon and eggs were purchased on four consecutive shopping trips (see [23, 28]).

	Shannon functional					
	Eggs					
Bacon	0	1	2	3	4	*r*
0	262.378	122.478	40.468	4.65702	0.0191661	0.999453
1	27.3702	23.502	18.8328	12.2212	4.0738	0.970398
2	5.38417	5.16918	4.87188	4.33981	3.23497	0.86233
3	1.25404	1.24078	1.22175	1.18545	1.09798	−0.0718339
4	0.613532	0.61028	0.605583	0.596516	0.574089	0.847078
	Likelihood functional					
	Eggs					
Bacon	0	1	2	3	4	*r*
0	258.603	118.489	40.1875	9.81096	2.90897	0.99991
1	30.4192	26.7046	18.5562	7.63744	2.68261	0.993516
2	6.02486	5.86333	5.34772	3.78732	1.97677	0.850168
3	1.32087	1.31294	1.28519	1.1694	0.911598	0.019223
4	0.631723	0.629903	0.623446	0.594872	0.520056	0.824691

Table 2 depicts the predictions based on Shannon functional, the likelihood functional and the Euclidean functional. In order to further condense the information, we have also calculated the correlation coefficient between each observed row and the corresponding expected one, reporting the obtained values in the last entry of each row of Table 2. The correlation coefficients are high for all the three functionals, which predict close values to the observed ones.

A closer inspection of Table 2 reveals that, as for the Bell Labs data set, the rows with the zeros are still the most problematic ones. However, the likelihood functional performs better than Shannon one: the predicted entries are closer to the real ones and the correlation coefficients are higher.

## 6 Some summary comments

This paper represents a contribution to the study of behavioral information recovery for self-organizing systems. The approach we proposed questions the use of traditional information recovery methods (see [13]), stressing the connections between adaptive behavior and causal entropy maximization (see [12]) in self organizing systems. This intuition can be formalized by implementing the procedure we propose, resting on the optimization of a class of entropic functionals under the constraints provided by the available information. Remarkably, other studies have presented results compatible with this view, i.e. that the real word is well approximated by maximum entropy ensembles where only partial information is used to reconstruct the entire system (see [10, 11, 29]).

The class of entropic functionals employed in this work is known as Cressie-Read family, which not only constitutes the analytical basis of our analysis but also represents a solution to the issue of solving ill-posed inverse problems by formally treating them as inference problems. Our results indicate that the performance of functionals constituting the CR family may vary significantly: in some cases, the likelihood functional (to the best of our knowledge, explicitly worked out here for the first time) provides the best performance; in others, it is outperformed by the Shannon functional. This indicates these two functionals are the ones making the best possible use of the available information, predicting the closest values to the observed ones.

In order to suggest applicability of our procedure, we have considered behavioral problems within the framework of network theory. The results we obtained not only indicate the effectiveness of our algorithm (applicable to univariate as well as bivariate data sets and for both *reproducing available data* and *predicting unavailable data*), but also demonstrate that networks are a useful way to present micro behavioral systems. In this context, the perspective proposed by our study can be enlarged by considering each node as a network on its own, a possibility which would simplify the task of modelling evolving networks, such as in the case of a growing economy, where a larger number of (adapting) nodes appear.

Given the importance of recovering dynamic economic behavioral information, a natural question arises about the continued use of traditional regularization information recovery methods as a solution basis for traditional pure and stochastic inverse type problems. For this reason, the next step is to extend the concept of adaptive-optimizing behavior and apply it (within the information theoretic framework) in the context of a range of micro economic settings, thus opening the promising perspective of turning the descriptive character of behavioral disciplines into a more quantitative one.

## Supporting Information

S1 Information We report and discuss a number of other cases of interest to which our methodology has been applied. (PDF)

## References

[1] Vardi Y. 1996 Network Tomography: Estimating Source-Destination Traffic Intensities From Link to Data. *Journal of the American Statistical Association* 91(433):365–377. 10.1080/01621459.1996.10476697

[2] Castro R., Coates M., Laing G., Nowak R. and Yu B. 2004 Network Tomography: Recent Developments. *Statistical Science* 19:499–517. 10.1214/088342304000000422

[3] Coates M. 2000 Network loss inference using unicast end-to-end measurement. *Proc. ITC Seminar on IP Traffic, Measurement, and Modeling* 28.

[4] Rubenstein D., Kurose J., Towsley D. 2002 Detecting shared congestion of flows via end-to-end measurement. *IEEE/ACM Transactions on Networking* 10(3):381–395. 10.1109/TNET.2002.1012369

[5] Annila A. and Salthe S. 2009 Economies Evolve by Energy Dispersal. *Entropy* 11:606–633. 10.3390/e11040606

[6] Georgescu-Roegen N. 1971 The Entropy law and the Economic process. Harvard University Press, Harvard.

[7] Willinger W., Alderson D. and Doyle J. 2009 Mathematics and the Internet: A Source of Enormous Confusion and Great Potential. *Journal of the American Mathematical Society* 56:586–599.

[8] Barabasi A.-L. 2012 The Network Takeover. *Nature Physics* 8:14–16. 10.1038/nphys2188

[9] Bargigli, L., Lionetta S. A. and Viaggiu, S. 2013. A Statistical Representation of Markets As complex Networks, http://arxiv.org/pdf/1307.0817v1.pdf.

[10] Mastrandrea R., Squartini T. and Garlaschelli D. 2014 Enhanced reconstruction of weighted networks from strengths and degrees. *New Journal of Physics* 16:043022 10.1088/1367-2630/16/4/043022

[11] Cimini, G., Squartini, T., Gabrielli A. and Garlaschelli, D. 2014. Estimating topological properties of weighted networks from limited information. http://arxiv.org/pdf/1409.6193.pdf. 10.1103/PhysRevE.92.040802 26565153

[12] Wissner-Gross A. D. and Freer C. E. 2013 Causal Entropic Forces. *Physical Review Letters* 110:168702 10.1103/PhysRevLett.110.168702 23679649

[13] Pressé S., Ghosh K., Lee J. and Dill K. 2013 Principles of Maximum Entropy and Maximum Caliber in Statistical Physics. *Reviews of Modern Physics* 85:1115–1141. 10.1103/RevModPhys.85.1115

[14] Raine A., Foster J. and Potts J. 2006 The New Entropy Law and the Economic Process. *Ecological complexity* 3:354–360. 10.1016/j.ecocom.2007.02.009

[15] Bound J., Jaeger D. A., Baker R. M. 1995 Problems with instrumental variables estimation when the correlation between the instruments and the endogenous explanatory variable is weak. *Journal of the American statistical association* 90(430):443–450. 10.2307/2291055

[16] Angrist, J., Krueger, A. B. 2001. Instrumental variables and the search for identification: From supply and demand to natural experiments. *No. w8456. National Bureau of Economic Research*.

[17] DiPrete T. A., Gangl M. 2004 Assessing bias in the estimation of causal effects: Rosenbaum bounds on matching estimators and instrumental variables estimation with imperfect instruments. *Sociological methodology* 34(1):271–310. 10.1111/j.0081-1750.2004.00154.x

[18] Cressie N. A. and Read T. 1984 Multinomial Goodness of Fit Tests. *Journal of the Royal Statistical Society, B* 46:440–464.

[19] Read T. and Cressie N. A. 1988 Goodness of Fit Statistics for Discrete Multivariate Data. Springer-Verlag, New York.

[20] Mittelhammer R. and Judge G. 2011 A family of empirical likelihood functions and estimators for the binary response model. *Journal of Econometrics* 164:207–217.

[21] Gorban A. N. and Karlin I. V. 2003 Family of Additive Entropy Functions out of Thermodynamic Limit. *Physical Review E* 67:016104 10.1103/PhysRevE.67.016104 12636561

[22] Judge G. and Mittelhammer R. C. 2012 An Information Theoretic Approach To Econometrics. Cambridge University Press, Cambridge.

[23] Cho, W. and Judge, G. 2014. An information theoretic approach to network tomography. *Applied Economics Letters*.

[24] Ziebart, B., Bagnell, J. and Dey, A. 2010. *Proceedings of an International Conference on Machine Learning* (Hiafa, Israel).

[25] Ziebart, B., Bagnell, J. and Dey, A. 2013. The principle of Maximum Causal Entropy for Estimating Interacting Processes. *IEEE Transactions For Information Theory* (in press).

[26] Cho W. and Judge G. 2006 Information Theoretic Solutions for Correlated Bivariate Processes. *Economic Letters* 7:201–207.

[27] Airoldi E. M. and Blocker A. W. 2013 Estimating latent processes on a network from indirect measurements. *Journal of the American Statistical Association* 108(501):149–164. 10.1080/01621459.2012.756328

[28] Crackel, R. and Flegal, J. M. 2014. Approximate Bayesian computation for a flexible class of bivariate beta distributions. http://arxiv.org/pdf/1402.1782.pdf.

[29] Squartini T. and Garlaschelli D. 2014 Stationarity, non-stationarity and early warning signals of economic networks. *Journal of Complex Networks*.

