# Supplementary material for: Information Recovery in Behavioral Networks
Source: PLoS One. 2015 May 6;10(5):e0125077. doi: 10.1371/journal.pone.0125077 (PMC4422444; doi:10.1371/journal.pone.0125077)
Supplement: S1 Information — (PDF) [file pone.0125077.s001.pdf]

Information Recovery In Behavioral Networks
Supporting Information

Tiziano Squartini,[1] Enrico Ser-Giacomi,[2] Diego Garlaschelli,[3] and George Judge[4]

[1] *Istituto dei Sistemi Complessi, Universitá di Roma "Sapienza", P.le A. Moro 5, 00185 Rome (Italy)*
[2] *IFISC (CSIC-UIB), Instituto de Física Interdisciplinar y Sistemas Complejos, Campus Universitat des les Illes Balears, E-07122 Palma de Mallorca (Spain)*
[3] *Lorentz Institute for Theoretical Physics, University of Leiden, Niels Bohrweg 2, 9506 Leiden (Netherlands)*
[4] *Graduate School and Giannini Foundation, University of California Berkeley, Berkeley, CA 94720 (United States)*

(Dated: April 22, 2015)

In the context of agent based modeling and network theory, we focus on the problem of recovering behavior-related choice information from origin-destination type data, a topic also known under the name of network tomography. As a basis for predicting agents' choices we emphasize the connection between adaptive intelligent behavior, causal entropy maximization and self-organized behavior in an open dynamic system. We cast this problem in the form of binary and weighted networks and suggest information theoretic entropy-driven methods to recover estimates of the unknown behavioral flow parameters. Our objective is to recover the unknown behavioral values across the ensemble analytically, without explicitly sampling the configuration space. In order to do so, we consider the Cressie-Read family of entropic functionals, enlarging the set of estimators commonly employed to make optimal use of the available information. More specifically, we explicitly work out two cases of particular interest: Shannon functional and the likelihood functional. We then employ them for the analysis of both univariate and bivariate data sets, comparing their accuracy in reproducing the observed trends.

PACS numbers: 89.75.Da; 02.50.Le; 89.65.Ef

I. SUPPORTING INFORMATION

A. Univariate data sets

As previously noted, eq. 6 induces a distribution on the ensemble of pathways. In other words, eq. 6 allows us to restate the problem of predicting the fluxes on origin-destination networks as a (more) general problem of statistical inference, where the unknown distribution on the pathways $\{p_c\}_{c=1}^{C}$ must be determined on the basis of partial information and represented by the conditions

$$\sum_c p_c = 1 \text{ and } \sum_c p_c Q_c^\alpha = \langle Q^\alpha \rangle,\ \alpha = 1 \dots M, \tag{I.1}$$

where the second equation in I.1 is nothing else than eq. 6, rephrased in more general terms (with Q_c^α replacing $A_{\alpha c}$ and $\langle Q^\alpha \rangle$ replacing r_α). Eq. 7 can thus be rewritten as

$$\mathcal{L} \equiv I(\mathbf{p}, \mathbf{q}, \gamma) - \theta_0 \left[\sum_c p_c - 1 \right] - \sum_\alpha \theta_\alpha \left[\sum_c p_c Q_c^\alpha - \langle Q^\alpha \rangle \right] \tag{I.2}$$

and the probability coefficients are obtained by solving the system

$$\frac{\partial \mathcal{L}}{\partial p_c} = 0,\ \forall\, c. \tag{I.3}$$

The resolution of the system I.3 gives us the desired coefficients $\{p_c\}_{c=1}^{C}$ as functions of the Lagrangean multipliers, $p_c = p_c(\vec{\theta})$, $\forall\, c$. Once found, the parametric probability coefficients must be substituted back into $\mathcal{L}$, in order to obtain a quantity which is a function of the unknowns solely: $\mathcal{L}(\vec{\theta})$. The last step in the procedure is the optimization of the function $\mathcal{L}(\vec{\theta})$, by finding the values of the parameters $\vec{\theta}^*$ which satisfy the condition

$$\left. \frac{\partial \mathcal{L}}{\partial \theta_i} \right|_{\vec{\theta}^*} = 0,\ \forall\, i. \tag{I.4}$$

For expository purposes, we explicitly demonstrate the analytical derivation of the Shannon functional for univariate data sets. In this case, the probability coefficients given by eq. I.3 have the expression

$$\frac{\partial \mathcal{L}}{\partial p_c} = 0 \Longrightarrow p_c = q_c \left(e^{-1+\theta_0+H_c} \right),\ \forall\, c \tag{I.5}$$

having defined $H_c \equiv \sum_\alpha \theta_\alpha Q_c^\alpha$. Our probability coefficients can be thus rewritten as

$$p_c = \frac{q_c e^{\sum_\alpha \theta_\alpha A_{\alpha c}}}{\sum_c q_c e^{\sum_\alpha \theta_\alpha A_{\alpha c}}},\ \forall\, c. \tag{I.6}$$

Substituting the analytical expression of p_c back into $\mathcal{L}$ produces a quantity which is solely function of the vector of unknown parameters $\vec{\theta}$ and the function to optimize with respect to the vector $\vec{\theta}$ becomes

$$\mathcal{L}(\vec{\theta}) = -\ln \left[\sum_c q_c \left(e^{\sum_\alpha \theta_\alpha A_{\alpha c}} \right) \right] + \sum_\alpha \theta_\alpha r_\alpha. \tag{I.7}$$

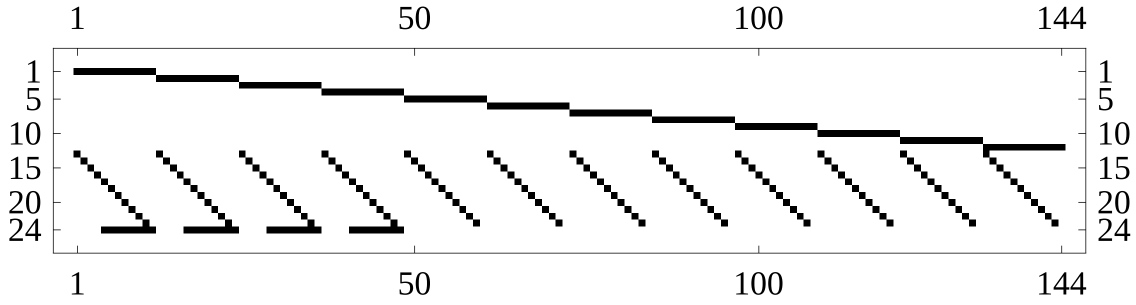

Figure A. Pictorial matrix representation of a local area network at the Information Networking Institute of Carnegie Mellon University (black squares represent ones, white squares represent zeros - see [27]), composed by twelve subnetworks, communicating via two routers (one with four subnetworks, the second one with the remaining eight subnetworks - the routers are linked via a single connection). The network topology we consider yields 24 observed aggregate traffic volumes and 144 origin-destination traffic volumes to be estimated.

B. A second worked-out example concerning univariate data sets

For completeness, we discuss a second example of traffic networks. The data set was collected at the Information Networking Institute of Carnegie Mellon University (see [27]) whose routing matrix is reported in **Figure A in S1 Information** The network topology we consider yields 24 observed aggregate traffic volumes and 144 origin-destination traffic volumes, observed every five minutes (473 points in time). This second dataset is larger than the first, allowing us to test the scalability of our approach.

The analysis of Carnegie University data is illustrated in **Figure B in S1 Information** Again, our method captures the chosen temporal trends, impliying that our procedure is applicable to problems with higher dimensionality. However, the results concerning Carnegie University data present some differences with respect to the Bell Labs ones.

Since a visual inspection of **Figure B in S1 Information** is not feasible, to quantify the agreement between our estimates and the observations we have calculated the correlation coefficient between the observed trends and the corresponding expected ones. The results for the Shannon functional are: $r = 0.994$ for the upper left panel (1st time point), $r = 0.991$ for the upper right panel (3rd time point), $r = 0.996$ for the middle left panel (80th time point), $r = 0.985$ for the middle right panel (190th time point), $r = 0.989$ for the bottom left panel (330th time point) and $r = 0.993$ for the bottom right panel (456th time point). The results for the likelihood functional are (in the same order): $r = 0.581$ (1st time point), $r = 0.595$ (3rd time point), $r = 0.703$ (80th time point), $r = 0.699$ (190th time point), $r = 0.693$ (330th time point) and $r = 0.701$ (456th time point).

Despite the rather high values of r, the strongly oscillatory character of the observed data set seems to have the effect of lowering the performance of our procedure: in fact, our estimations predict a "smoother" behavior than that of real data which, on the other hand, appear much more irregular (see lowest panels of **Figure B in S1 Information**). As for the Bell Labs data set, the net result is that high values of traffic data are well estimated while the lower ones (included the zero ones) are generally overestimated.

Quite surprisingly, even the differences characterizing the performances of the two functionals are larger than for the Bell Labs data set: this time the best result (witnessed by the higher correlation coefficients for all the time points) is obtained by the Shannon functional which seems to better follow the irregular observed trends: the predictions obtained by the likelihood functional, in fact, show flat regions which in turn have the effect of lowering the numerical correlation value.

C. Bivariate data sets

For bivariate problems, the CR family of functionals becomes

$$I(\mathbf{p},\mathbf{q},\gamma)=\frac{1}{\gamma(\gamma+1)}\sum_j\sum_k p_{jk}\left[\left(\frac{p_{jk}}{q_{jk}}\right)^{\gamma}-1\right] \tag{I.8}$$

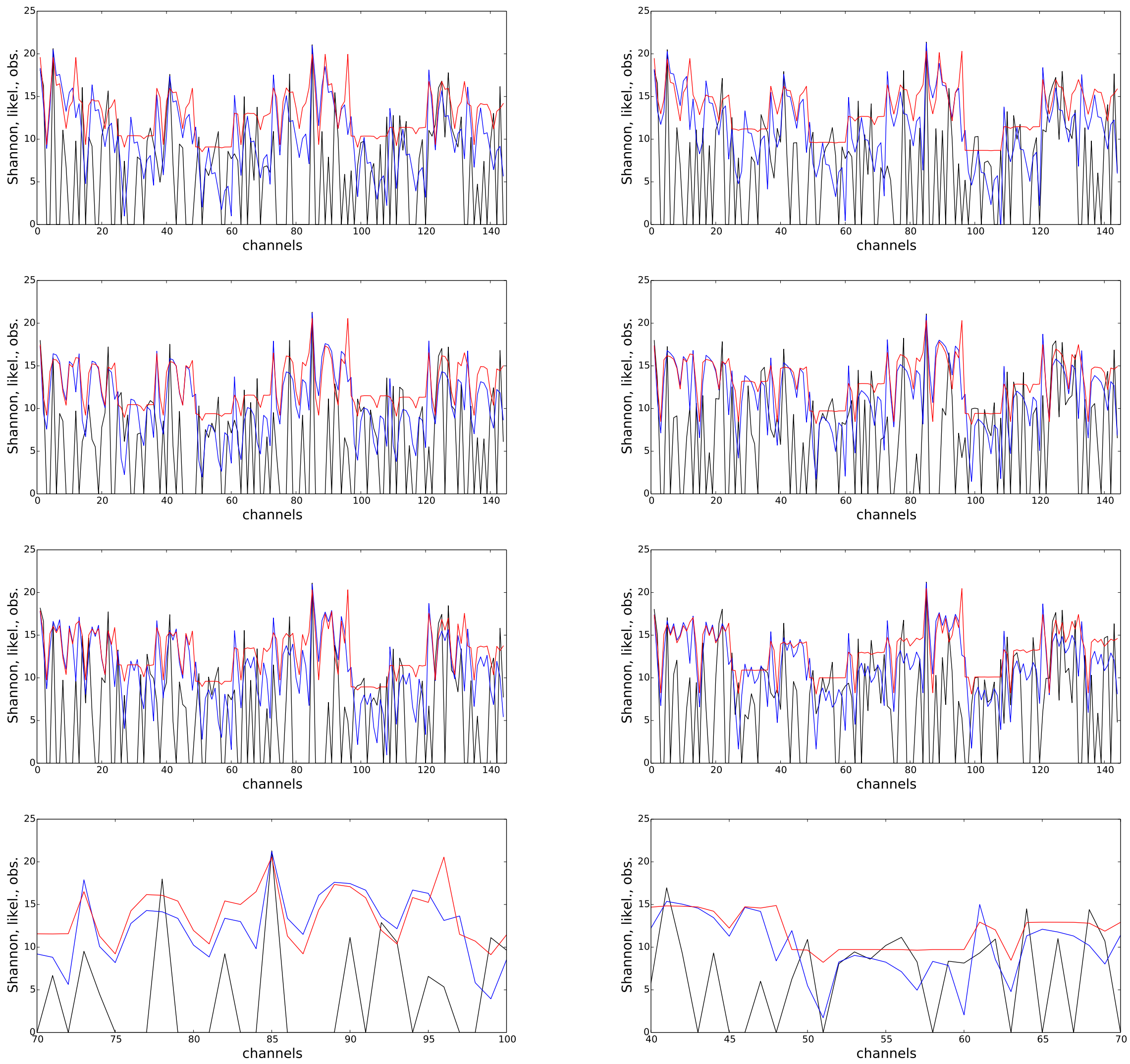

Figure B. Analysis of Carnegie University data corresponding to six chosen time points. The number of the channel is reported on the x-axis. Observed and estimated $\mathbf{x}$ are reported on the y-axis (logarithmic scale). Colors referes to: observed data (black trend), our estimation based on Shannon functional (blue trend), our estimation based on the likelihood functional (red trend). The lowest panels show a zoomed region of the "channel plots" corresponding to the 80th and 190th time points.

j and k respectively indicating the row and column index of the probability matrix $\mathbf{P}$ to be estimated and of the prior, bivariate one $\mathbf{Q}$. The constraints are now represented by the conditions

$$\sum_k p_{jk} = 1,\ \forall\, j \ \text{ and } \ \sum_j x'_j p_{jk} = y'_k,\ \forall\, k. \tag{I.9}$$

For bivariate problems, the number of multipliers rises, since the required number of normalization conditions equals the number of matrix rows. Thus, in order to correctly implement our approach, two vectors $\vec{\alpha}$ and $\vec{\beta}$ must be considered. Constraining equation I.8 for bivariate data sets (and again for Shannon entropy) leads to

$$I\left(\mathbf{p}, \frac{1}{C}, 0\right) = \sum_j \sum_k p_{jk} \ln p_{jk} + \ln C - \sum_j \beta_j \left(\sum_k p_{jk} - 1\right) - \sum_k \alpha_k \left(\sum_j p_{jk} x'_j - y'_k\right) \tag{I.10}$$

Table A. Precint-level data of Louisiana's 5th CD elections (see [23]).

	Rep.	Dem.	Ind.1	Ind.2	Abst.	Total
White	–	–	–	–	–	1158
Black	–	–	–	–	–	222
Other	–	–	–	–	–	31
Total	963	207	28	17	196	1411

and maximizing it with respect to p_{jk} implies that the functional form of our coefficients is

$$p_{jk} = \frac{e^{\alpha_k x'_j}}{\sum_k e^{\alpha_k x'_j}}, \; \forall\, j, k; \tag{I.11}$$

by substituing back into $\mathcal{L}$ we get

$$\mathcal{L}(\vec{\alpha}) = -\sum_j \left(\ln\left[\sum_k e^{\alpha_k x'_j} \right] + \sum_k \alpha_k x'_j \right). \tag{I.12}$$

Similar results are obtained for the other functionals.

D. A second worked-out example concerning bivariate data sets

The second bivariate data set we discuss comes from an application in political science and concerns voter behavior and candidate choice (as reported in **Table A in S1 Information** - see [26]). The result of the application of our method to the elections percentages is shown in **Table B in S1 Information**.

Since privacy issues prevent the percentage of people voting for a given candidate from being available, the second bivariate data set we analyzed provides only aggregate data about the elections results: the single matrix entries are thus missing. Nonetheless, our method provides a prediction of the unknown entries, by adopting the same procedure used for the "eggs and bacon" problem. As can be seen from table **Table B in S1 Information**, Shannon functional and the likelihood functional give compatible estimates of the voting percentages: this similarity is effectively summed up by the "global" Pearson correlation coefficient between the Shannon expected matrix and the likelihood expected matrix (both treated as an unique vector of numbers), equal to 0.988716. It should be noted, however, that significative differences can be observed for the percentages referring to the independent candidates. Nonetheless, when interpreted in the light of the previous results, these differences carry an important information, signalling that independent candidates true percentages are, probably, not only the lowest ones, but even compatible with zero.

Table B. Estimated precint-level percentages of Louisiana's 5th CD elections (see [23]).

	Shannon functional					
	Rep.	Dem.	Ind.1	Ind.2	Abst.	Total
White	877.555	144.824	0.968424	0.0422665	134.611	1158
Black	78.5616	55.6173	21.2953	11.6828	54.843	222
Other	6.88327	6.55916	5.73627	5.27497	6.54634	31
	Likelihood functional					
	Rep.	Dem.	Ind.1	Ind.2	Abst.	Total
White	865.704	141.143	12.2831	6.89041	131.831	1158
Black	89.4101	58.4307	10.9359	6.44502	56.7707	222
Other	7.7549	7.41397	4.77993	3.66401	7.38656	31
Total	963	207	28	17	196	1411

ACKNOWLEDGEMENTS

TS acknowledges support from the Italian PNR project CRISIS-Lab.

ESG acknowledges support from the European Commission Marie-Curie ITN program (FP7-320 PEOPLE-2011-ITN) through the LINC project (no. 289447).

DG acknowledges support from the Dutch Econophysics Foundation (Stichting Econophysics, Leiden, the Netherlands). This work was also supported by the project MULTIPLEX (contract 317532) and the Netherlands Organization for Scientific Research (NWO/OCW). The funders had no role in study design, data collection and analysis, decision to publish, or preparation of the manuscript.

---

[1] Vardi, Y. 1996. Network Tomography: Estimating Source-Destination Traffic Intensities From Link to Data. *Journal of the American Statistical Association* 91(433):365-377.

[2] Castro, R., Coates, M., Laing, G., Nowak, R. and Yu, B. 2004. Network Tomography: Recent Developments. *Statistical Science* 19:499-517.

[3] Coates, M. 2000. Network loss inference using unicast end-to-end measurement. *Proc. ITC Seminar on IP Traffic, Measurement, and Modeling* 28.

[4] Rubenstein, D., Kurose, J., Towsley, D. 2002. Detecting shared congestion of flows via end-to-end measurement. *IEEE/ACM Transactions on Networking* 10(3):381-395.

[5] Annila, A. and Salthe, S. 2009. Economies Evolve by Energy Dispersal. *Entropy* 11:606-633.

[6] Georgescu-Roegen, N. 1971. The Entropy law and the Economic process. Harvard University Press, Harvard.

[7] Willinger, W., Alderson, D. and Doyle, J. 2009. Mathematics and the Internet: A Source of Enormous Confusion and Great Potential. *Journal of the American Mathematical Society* 56:586-599.

[8] Barabasi, A.-L. 2012. The Network Takeover. *Nature Physics* 8:14-16.

[9] Bargigli, L., Lionetta S. A. and Viaggiu, S. 2013. A Statistical Representation of Markets As complex Networks, http://arxiv.org/pdf/1307.0817v1.pdf.

[10] Mastrandrea, R., Squartini, T. and Garlaschelli, D. 2014. Enhanced reconstruction of weighted networks from strengths and degrees. *New Journal of Physics* 16:043022.

[11] Cimini, G., Squartini, T., Gabrielli A. and Garlaschelli, D. 2014. Estimating topological properties of weighted networks from limited information. http://arxiv.org/pdf/1409.6193.pdf.

[12] Wissner-Gross, A. D. and Freer, C. E. 2013. Causal Entropic Forces. *Physical Review Letters* 110:168702.

[13] Pressé, S., Ghosh, K., Lee, J. and Dill, K. 2013. Principles of Maximum Entropy and Maximum Caliber in Statistical Physics. *Reviews of Modern Physics* 85:1115-1141.

[14] Raine, A., Foster, J. and Potts, J. 2006. The New Entropy Law and the Economic Process. *Ecological complexity* 3:354-360.

[15] Bound, J., Jaeger, D. A., Baker, R. M. 1995. Problems with instrumental variables estimation when the correlation between the instruments and the endogenous explanatory variable is weak. *Journal of the American statistical association* 90(430):443-450.

[16] Angrist, J., Krueger, A. B. 2001. Instrumental variables and the search for identification: From supply and demand to natural experiments. *No. w8456. National Bureau of Economic Research.*

[17] DiPrete, T. A., Gangl, M. 2004. Assessing bias in the estimation of causal effects: Rosenbaum bounds on matching estimators and instrumental variables estimation with imperfect instruments. *Sociological methodology* 34(1):271-310.

[18] Cressie, N. A. and Read, T. 1984. Multinomial Goodness of Fit Tests. *Journal of the Royal Statistical Society, B* 46:440-464.

[19] Read, T. and Cressie, N. A. 1988. Goodness of Fit Statistics for Discrete Multivariate Data. Springer-Verlag, New York.

[20] Mittelhammer, R. and Judge, G. 2011. A family of empirical likelihood functions and estimators for the binary response model. *Journal of Econometrics* 164:207-217.

[21] Gorban, A. N. and Karlin, I. V. 2003. Family of Additive Entropy Functions out of Thermodynamic Limit. *Physical Review E* 67:016104.

[22] Judge, G. and Mittelhammer, R. C. 2012. An Information Theoretic Approach To Econometrics. Cambridge University Press, Cambridge.

[23] Cho, W. and Judge, G. 2014. An information theoretic approach to network tomography. *Applied Economics Letters*, doi:10.1080/13504851.2013.866199.

[24] Ziebart, B., Bagnell, J. and Dey, A. 2010. *Proceedings of an International Conference on Machine Learning* (Hiafa, Israel).

[25] Ziebart, B., Bagnell, J. and Dey, A. 2013. The principle of Maximum Causal Entropy for Estimating Interacting Processes. *IEEE Transactions For Information Theory* (in press).

[26] Cho, W. and Judge, G. 2006. Information Theoretic Solutions for Correlated Bivariate Processes. *Economic*

Letters 7:201-207.
[27] Airoldi, E. M. and Blocker, A. W. 2013. Estimating latent processes on a network from indirect measurements. *Journal of the American Statistical Association* 108(501):149-164.
[28] Crackel, R. and Flegal, J. M. 2014. Approximate Bayesian computation for a flexible class of bivariate beta distributions. http://arxiv.org/pdf/1402.1782.pdf.
[29] Squartini, T. and Garlaschelli, D. 2014. Stationarity, non-stationarity and early warning signals of economic networks. *Journal of Complex Networks*, doi:10.1093/comnet/cnu012.
